# Treatment-Related Fluctuation in Guillain-Barre Syndrome With the Acute Motor-Sensory Axonal Neuropathy (AMSAN) Variant: A Case Report

**DOI:** 10.7759/cureus.65201

**Published:** 2024-07-23

**Authors:** Elliot Hernandez, David Dominguez, Raul Medina-Rioja, Victoria Martínez-Angeles, Juan Carlos López-Hernández

**Affiliations:** 1 Emergency Department, Instituto Nacional de Neurologia y Neurocirugia Manuel Velasco Suárez, Mexico City, MEX; 2 Neurology Department, Instituto Nacional de Neurologia y Neurocirugia Manuel Velasco Suárez, Mexico City, MEX; 3 Neuropsychiatry Department, Instituto Nacional de Neurologia y Neurocirugia Manuel Velasco Suárez, Mexico City, MEX; 4 Neuromuscular Diseases Department, Instituto Nacional de Neurologia y Neurocirugia Manuel Velasco Suárez, Mexico City, MEX

**Keywords:** amsan variant, case report, ayahuasca, treatment-related fluctuation, guillain-barre syndrome

## Abstract

We present the case of a 60-year-old male patient with Guillain-Barré syndrome (GBS) who experienced treatment-related fluctuations (TRF) with a history of ayahuasca consumption. The patient presented to the neurological emergency department without a history of infection (upper respiratory tract or diarrhea) or vaccination in the past four weeks, but 14 days prior, the patient had consumed ayahuasca. Upon admission, the patient exhibited progressive weakness in all four limbs, with no cranial nerve involvement, a muscle strength Medical Research Council (MRC) score of 36/60, and generalized areflexia. Cerebrospinal fluid analysis showed slightly elevated protein levels at 50 mg/dL and a cell count of 2 (lumbar puncture was performed three days after the onset of symptoms). Neurophysiological studies met the criteria for the acute motor-sensory axonal neuropathy (AMSAN) variant. A diagnosis of GBS was established, Brighton criteria grade 1. The patient received treatment with intravenous human immunoglobulin, resulting in improvement with an MRC score of 48/60 at discharge. However, on day 10, he returned with worsening muscle strength (MRC score of 20/60), necessitating ventilatory support. TRF was considered, and retreatment with human immunoglobulin was initiated.

## Introduction

Guillain-Barré syndrome (GBS) is the leading cause of acute flaccid paralysis worldwide. It is triggered by exposure to an environmental agent in immunologically susceptible individuals, resulting in an aberrant immune response directed against peripheral nerves. In 70% of cases, a history of upper respiratory tract infection or gastrointestinal infection (diarrhea) can be documented through clinical history. In Mexico, as in other Latin American countries, the most common infectious agent is Campylobacter jejuni, which causes diarrhea in the population and is associated with axonal electrophysiological variants (AMAN: acute motor axonal neuropathy, AMSAN: acute motor-sensory axonal neuropathy) [1,2].

Intravenous human immunoglobulin (IVIg) and plasma exchange are equally effective therapies for the treatment of GBS [2]. Nine percent of patients treated with IVIg (2 g/kg divided over five days) exhibit treatment-related fluctuation (TRF), which is defined by two clinical scenarios: 1) improvement in the GBS disability scale by at least one grade or more than 5 points in the Medical Research Council (MRC) score within four weeks, followed by a decrease in the MRC score by more than 5 points or worsening in the GBS disability scale by at least one grade; and 2) stabilization of the clinical course for more than a week, followed by a worsening of more than 5 points in the MRC score or by at least one grade in the GBS disability scale. TRFs predominantly occur in patients with the acute inflammatory demyelinating polyneuropathy (AIDP) variant, few cases have been reported with the AMSAN variant [3].

Ayahuasca is an ancestral plant used by native peoples of South America and some indigenous communities in Mexico for spiritual rituals. The beverage prepared with ayahuasca, given to participants in these rituals, presents some side effects such as psychosis, nausea, vomiting, headache, etc. [4].

The objective of this manuscript is to report a case of GBS with the AMSAN variant that presented fluctuations related to treatment with IVIg, following the ingestion of ayahuasca beverage.

## Case presentation

A 60-year-old male patient, with no history of previous infection (upper respiratory tract or diarrhea) or vaccination in the last four weeks, attended a healing ritual 14 days prior to his illness where he consumed four bottles of ayahuasca (200 mL each). He experienced side effects (visual hallucinations, nausea, and vomiting), which resolved within 24 hours. Three days before admission to the neurological emergency department, he began experiencing paresthesia in both hands and feet and progressive weakness in both lower and upper limbs, leading to an inability to walk. Upon admission, the patient had stable vital signs, normal mental functions, and no cranial nerve abnormalities. The motor system showed decreased tone and muscle strength with an MRC score of 36/60 points (deltoids, biceps, wrist extensors, iliopsoas, quadriceps, and anterior tibialis), with generalized areflexia. General exams (complete blood count, blood chemistry, electrolytes, liver function tests) were unremarkable; cerebrospinal fluid analysis from lumbar puncture showed proteins at 50 mg/dL and a cell count of 2; the detection of antigangliosides and markers or culture for isolation of Campylobacter jejuni was not carried out due to the lack of resources. A neuroconduction study met Rajabally's criteria for the AMSAN variant (Table 1). A diagnosis of GBS was made, with a GBS disability scale score of 4 at admission, and Brighton level 1 diagnostic certainty [5]. The patient received treatment with intravenous immunoglobulin at 2 g/kg divided over five days and was discharged home six days after admission with improved muscle strength (MRC score of 48/60) and a GBS disability scale score of 3 points.

**Table 1:** Neuroconduction study of motor and sensory nerves

	Right Nerves				Left Nerves			
	Distal latency (ms)	Conduction velocity (m/s)	CMAP distal (mV)	CMAP proximal (mV)	Distal latency (ms)	Conduction velocity (m/s)	CMAP distal (mV)	CMAP proximal (mV)
Motor nerves								
Median	4.3	47	2.9	1.7	5.1	44	3.5	2.3
Ulnar	2.9	51	0.8	0.5	3.0	53	1.7	1.2
Tibial	4.1	45	1.3	0.8	4.4	46	1.8	1.3
Peroneal	4.4	48	0.9	0.4	4.1	45	1.2	0.9
Sensory nerves			SNAP (µV)				SNAP (µV)	
Median	NR	NR	NR	-------	NR	NR	NR	-------
Ulnar	NR	NR	NR	-------	NR	NR	NR	-------
Sural	2.1	70	13.6	-------	1.6	82	16.1	-------
CMAP: compound muscle action potential; SNAP: sensory nerve action potential; NR: no response; mV: millivolts; μV: microvolts; m/s: meters/second. *F waves (median, ulnar, tibial and peroneal), no response.								

The patient was readmitted to the emergency department 10 days later due to progressive muscle weakness in all four limbs, accompanied by respiratory difficulty. On this admission, he had a saturation of 80%, respiratory rate of 40 breaths per minute, heart rate of 110 beats per minute, and an MRC score of 20 points, necessitating invasive mechanical ventilation. Complications during orotracheal intubation led to a cardiorespiratory arrest (midazolam and rocuronium were used intravenously as anesthetic agents), requiring two cycles of cardiopulmonary resuscitation, after which he achieved a heart rate of 110 bpm in sinus rhythm and required vasopressor support. Due to the worsening muscle strength compared to the previous discharge, it was decided to treat him as a patient with treatment-related fluctuation in GBS, and intravenous immunoglobulin was administered again at 2 g/kg divided over five days. Two days after his readmission, a neuroconduction study was performed, which was reported compatible with the AMSAN variant. Unfortunately, the patient developed hypoxic/ischemic encephalopathy, and after a prolonged hospital stay, he was discharged in a state of minimal consciousness.

## Discussion

The most commonly used treatments for GBS are plasma exchange (four to five sessions) or IVIg at 2 g per kg [6]. Both therapies report treatment-related fluctuation (TRF) in 6-9% of patients; however, this condition is better described with IVIg. These reports are conducted in European populations, and there is no information on Mexico or other Latin American countries [7].

TRF is defined as a worsening of muscle strength after treatment, specifically a decrease of at least 5 points on the MRC scale or an increase of one grade on the GBS disability scale [4]. Limited information exists about the clinical and paraclinical characteristics of patients with TRF. The literature indicates no gender or age predominance, nor any relation to a specific electrophysiological variant [4,7]. One study reported that patients with TRF had fewer days of symptom evolution at admission, a higher frequency of cranial nerve involvement, and lower MRC scores [4]. Another study reported that a history of infectious mononucleosis was more frequent in patients with TRF [6]. In the case of our patient, he was admitted to the emergency department with three days of symptom evolution and no cranial nerve involvement, without an infectious history, but with a recent history of ayahuasca consumption.

The timing of TRF onset after GBS treatment is highly variable, ranging from two to 25 days. A patient may experience more than one episode of TRF, provided it occurs within two months. Beyond this period, the patient is classified as having chronic inflammatory demyelinating polyneuropathy (CIDP), and previous episodes would be considered acute-onset CIDP [8]. Patients with TRF should be retreated with the same therapy. For IVIg, a regimen of 2 g/kg is prescribed again, which incurs significant costs for patients or healthcare institutions in developing countries. In the case of our patient, TRF occurred on day 10, with worsening muscle strength requiring invasive mechanical ventilation support, for which he was re-treated with IVIg.

Ayahuasca is an ancestral beverage used by indigenous communities in several South American countries, as well as some indigenous communities in Mexico, for spiritual healing rituals, which also attract non-indigenous individuals. Ayahuasca is prepared as a tea using the plant Banisteriopsis caapi, which grows as a liana, along with leaves from the shrub Psychotria viridis. The main compounds of Banisteriopsis caapi are β-carbolines, such as harmine, tetrahydroharmine (THH), and harmaline, while the leaves of Psychotria viridis are rich in the hallucinogenic tryptamine N, N-dimethyltryptamine (DMT). Although studies in psychiatric populations have shown benefits in patients with major depressive disorder or substance use disorders, ayahuasca is not yet approved as a treatment for these conditions. The main immediate and transient side effects of ayahuasca consumption are hallucinations, vomiting, nausea, and headache [4,9].

GBS typically occurs after an infectious disease. In countries such as Mexico, the main infectious agents (bacterial or viral) are those related to diarrhea, and patients are exposed to these agents through contaminated food or liquids [2]. The active principles of the plants used in ayahuasca act at the central nervous system level and do not cause peripheral nerve damage [4]. However, a study reported that the consumption of ayahuasca transiently reduces CD4 and CD3 lymphocyte levels compared to controls [9]. CD4 lymphocytes are responsible for organizing the immune response (cellular and humoral) against infectious agents (bacteria and viruses), leaving memory cells localized in the epithelia (respiratory, gastrointestinal) their reduction levels predispose individuals to infections [10]. GBS is an inflammatory polyneuropathy that is triggered after an infection in 70% of cases. Its immunopathology has demonstrated a reduction in CD4 lymphocyte levels [11]. In the case of our patient, we theorize that GBS was triggered by an infectious agent obtained through the ayahuasca bottles (possibly from the water used or poor preservation of the bottles), combined with a possible transient reduction in CD4 lymphocyte levels. The authors acknowledge that more information is needed to establish a relationship between ayahuasca consumption and GBS. However, as the practice of attending healing rituals where ayahuasca is consumed becomes more frequent among the non-indigenous population [4], we recommend that individuals participating in these rituals verify how ayahuasca is prepared and stored.

## Conclusions

We reported the case of a patient diagnosed with GBS (the AMSAN variant), who had a history of ayahuasca consumption. The patient exhibited TRFs following the administration of intravenous human immunoglobulin. Limited information exists on the clinical characteristics and factors related to TRFs occurring in 9% of GBS cases. The use of ayahuasca in healing rituals is becoming increasingly common among non-indigenous populations. Although the active properties of ayahuasca do not cause damage to peripheral nerves, the water used in its preparation and storage may contain infectious agents associated with GBS.
